# Systematic Development Strategy for Smart Devices Based on Shape-Memory Polymers

**DOI:** 10.3390/polym9100496

**Published:** 2017-10-10

**Authors:** Andrés Díaz Lantada

**Keywords:** shape-memory polymers, additive manufacturing, 3D printing, 4D printing, “smart” materials and structures, shape-memory composites, advanced actuators

## Abstract

Shape-memory polymers are outstanding “smart” materials, which can perform important geometrical changes, when activated by several types of external stimuli, and which can be applied to several emerging engineering fields, from aerospace applications, to the development of biomedical devices. The fact that several shape-memory polymers can be structured in an additive way is an especially noteworthy advantage, as the development of advanced actuators with complex geometries for improved performance can be achieved, if adequate design and manufacturing considerations are taken into consideration. Present study presents a review of challenges and good practices, leading to a straightforward methodology (or integration of strategies), for the development of “smart” actuators based on shape-memory polymers. The combination of computer-aided design, computer-aided engineering and additive manufacturing technologies is analyzed and applied to the complete development of interesting shape-memory polymer-based actuators. Aspects such as geometrical design and optimization, development of the activation system, selection of the adequate materials and related manufacturing technologies, training of the shape-memory effect, final integration and testing are considered, as key processes of the methodology. Current trends, including the use of low-cost 3D and 4D printing, and main challenges, including process eco-efficiency and biocompatibility, are also discussed and their impact on the proposed methodology is considered.

## 1. Introduction

Shape-memory polymers (SMPs) are active or “smart” materials that present a mechanical response to external stimuli, normally changes in surrounding temperatures. Although other types of stimuli such as light, water or chemicals, can promote shape-memory effects in polymers, we focus here on thermally activated shape-memory polymers, as they are the most common ones. When these materials are heated above their “activation” temperature (*T*_act_), typically corresponding to glass (*T*_g_) or melting transitions (*T*_m_), a radical stiffness change takes and the SMPs change from a rigid to an elastic state, which in some cases allows deformations of up to 400%. After being manipulated and deformed, if the material is cooled down with the imposed deformation, this structure is “frozen” and returns to a rigid but “unbalanced” state. This process is usually referred to as “shape-memory training” process. When the material is once again heated above its “activation temperature” (normally corresponding to glass transitions temperatures), it returns to its initial non-deformed state. In many cases, the training and actuation cycle can be repeated numerous times without any or just slight degradation to the polymer and most suppliers can formulate different materials with typical values of activation temperatures between −50 °C and 250 °C, according to the desired final applications. Among the polymers developed with remarkable shape-memory properties, some of the most important are epoxy resins, polyurethane resins, cross-linked polyethylene, diverse styrene-butadiene copolymers, and other formulations described in previous foundational reports and studies [1,2,3,4].

The more traditional shape-memory polymers are “smart” materials that possess thermo-mechanical or electro-thermo-mechanical couplings and an ability to recover from high deformations, (much greater than that of shape-memory metal alloys and other types of shape-memory materials), which combined with their lower density and cost has encouraged the design of numerous applications. Their properties permit applications in the manufacture of sensing devices or actuators, particularly for the aeronautic, automobile and medical industries. Their recent proposals for medical use, which benefit from the enhanced biocompatibility of several polymers, have been examined previously [5,6]. However, if the development of new and more demanding applications is to be encouraged, especially for the aforementioned advanced industries, the synthesis, processing, modeling, design, prototyping, characterization and environmental responses of these recent materials need to be examined closely [1,2,4,7,8,9,10,11].

Even if the initial studies in the field of shape-memory polymers mainly focused on thermally activated polymers, more recent developments have detailed the activation of geometrical changes in shape-memory polymers due to other external cues or environmental phenomena, which promotes the versatility of these materials and their applicability to a wider set of industries and contexts. To cite some foundational examples, water-responsive shape-memory polymers and thermo-moisture responsive SMPs have been developed and characterized [12,13]. Chemical reactions, including biodegradation processes [14] or thermo-chemical responses [15], may also help to control the activation of SMPs. In some cases, these responses may also promote innovative 4D printing applications [16] or may be connected to step-by-step, to triple- or multi-shape memory effects and to reversible SMPs [17,18,19] for avant-garde smart devices. The reversibility of shape-memory polymers controlled by electro-magnetic fields has been also recently validated, which also opens the horizon of applications.

As regards the development of final applications, more conventional thermally activated shape-memory polymers require the design of a heating system, which normally affects final device size and the mechanical performance of the active material. Alternative options for SMP-based actuators may rely on the use of the more recently discovered triggering stimuli (i.e., water immersion, pH changes, application of UV light, electro-magnetic fields, etc.), which may provide benefits in terms of final device size and homogeneity of activation, while presenting new design challenges linked to the subsystems required for managing the application of such stimuli.

Present study presents a review of challenges and good practices, leading to a straightforward methodology or integration of strategies, for the development of “smart” actuators based on shape-memory polymers. An engineering design approach, based on the “Conceive–Design–Implement–Operate” (CDIO) cycle, is proposed and main challenges, potential solutions and current trends of research, linked to the different stages of the product’s lifecycle, are discussed in the following sections. The combination of computer-aided design, computer-aided engineering and additive manufacturing technologies, with special impact on the design and implementation stages, is analyzed and applied to the complete development of interesting shape-memory polymer-based actuators. Aspects such as geometrical design and optimization, development of the activation system, selection of the adequate materials and related manufacturing technologies, training of the shape-memory effect, final integration and testing are considered, as key processes of the methodology. Recent technological breakthroughs, including the use of low-cost 3D and 4D printing, and main present concerns for several potential applications in high tech industries, including process eco-efficiency and biocompatibility, are also discussed. Their impact on the proposed methodology is considered and a final summary of good-practices is provided.

In order to present the most relevant issues for the straightforward development of “smart” devices based on shape-memory polymers, a couple of complete developments are used as cases of study in present report. The first (bio-)device used as case of study is an active catheter end resembling a micro-gripper. The second case of study is an active annuloplasty ring for the potential and progressive treatment of mitral valve insufficiency. Main materials and resources have been detailed in previous works by our team [10,20,21]. In short, we combine computer-aided design and finite element modeling resources, additive manufacturing technologies for the direct fabrication of geometries using SMPs, rapid tooling techniques for casting of enhanced SMPs into rapid molds and infrared thermography for secure testing of the final devices. These materials and methods can be applied to the design and development of several smart materials and structures and are very well suited for supporting the straight-forward development of “smart” actuators based on shape-memory polymers, as the review of challenges and good practices presented in the following Section further details.

## 2. Review of Challenges and Good Practices for the Development of “Smart” Actuators Based on Shape-Memory Polymers: Towards a Straightforward Development Methodology

The field of shape-memory polymers and composites has greatly evolved in the last two decades, thanks to the efforts and talent of several chemists, physicists, materials scientists and engineers, whose research and development activities, linked to the synthesis and processing of these materials, has helped to generate a whole new family of “smart” materials ready for application [22]. However, the incorporation of shape-memory polymers as actuators in complex engineering systems is still not so direct and can benefit from the use of a systematic methodology and from the utilization of validated good-practices and common solutions for typical challenges. Main challenges present in the different stages of the development process of novel actuators based on shape-memory polymers, together with a discussion of good-practices and validated solutions are detailed in this Section.

### 2.1. Challenges and Solutions Linked to the Conceptual Stage

The use of “smart” materials as actuators in engineering systems is usually linked to a need for complexity, size and weight reduction, as the material itself serves as transducer, hence reducing the number of auxiliary components, minimizing the possibility of failure and sinking the production costs. Nevertheless, due to the steadily growing number of novel families of “smart” materials and to the recent discovery of some of the most promising ones, including shape-memory polymers, it is important to make some preliminary considerations, during the conceptual stage of the development of any new “smart” actuator. Debate questions for creativity promotion during the initial stages include:

(1) Would the device benefit from the use of a “smart” material?
(2) Would the “smart” material help to reduce complexity, size, weight or costs?
(3) Would the “smart” material help to increase overall reliability?
(4) Would a SMP outperform other “smart” materials in the proposed application?

**Figure 1:**
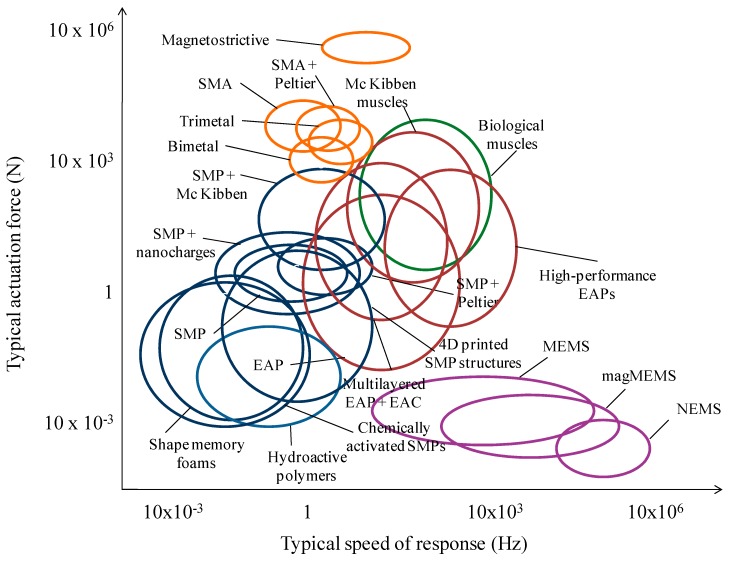
Usual actuation force vs. speed of response for actuators based on “smart” materials. Abbreviations: EAC, electroactive (normally piezoelectric) ceramics; EAP, electroactive polymers; SMP, shape-memory polymers; SMA, shape-memory alloys; MEMS, microelectromechanical systems; magMEMS, Magnetic microelectromechanical systems; NEMS, nanoelectromechanical systems. Adapted and updated from study by our team [23].

The use of material selection charts or “Ashby” diagrams, but specifically adapted to the properties of “smart” materials as actuators [23], such as the one developed by our team and included in Figure 1, may prove to be a powerful resource for these initial selection tasks. Aspects such as the actuation force, the attainable displacements, the speed of response and the bandwidth are key characteristics of engineering actuators and should be considered (and quantitatively specified at the very initial stages of the development), when selecting a family of “smart” materials as potential source of solutions for a concrete device.

As can be appreciated in Figure 1, with typical actuation forces in the range of 1–100 N and with common actuation speeds of 0.5–10 s/cycle, shape-memory polymers are not among the most powerful or rapid families of “smart” materials. In addition, once activated, they normally perform just one actuation cycle, although interesting advances linked to triple-shape memory polymers and composites [16,17,18,19,24,25] and novel possibilities enabled thanks to the concept of 4D printing [26] are helping to promote the number of attainable actuation steps. It is also important to note that, once an actuation cycle has been completed, the shape-memory polymer can be trained again and new cycles can be performed, although they are not typically aimed at repetitive actuation steps.

However, shape-memory polymers stand out for the extremely large deformations attainable (usually up to a 400%) and for the consequent shape changes achievable during actuation. In addition, they can be manufactured using a wide set of technologies, including casting, machining and 3D/4D printing, which promotes geometrical freedom, and integration into complex devices. Being polymeric materials, the use of additives can also help to adapt their performance to the proposed application, almost in a personalized way.

Another key aspect during the conceptual stage is linked to an adequate selection of material suppliers and to the need of exhaustive information, not only for adequately selecting the shape-memory polymer(s) to be used, but also as input for properly performed design tasks. The fact with most recently developed families of “smart” materials, and shape-memory polymers are not an exception, is that most suppliers provide only partial, and typically the most positive, information regarding the actuation capabilities of the material. Relevant aspects including creep behavior, stress relaxation, fatigue performance, physical or chemical ageing, water absorption, among other key characteristics for a shape-memory polymer to be used for a final demanding application, are not commonly known (or provided) by developers. Consequently, exhaustive characterization using preliminary probes is a very advisable practice during the conceptual stage to validate if a candidate material may perform as expected and to obtain relevant information for the subsequent design stage.

### 2.2. Challenges and Solutions Linked to the Design Stage

The design phase of actuators based on shape-memory polymers includes special considerations, which may help to reach more efficient and effective solutions and to minimize problems in the subsequent development stages, if adequately taken into account. Different discussion questions for a more direct design (helping designers to avoid steps taken forward and back again typical from trial and error approaches) may include:

(1) Should we use a shape-memory material for the whole structure or just for active zones?
(2) Which are the typical geometries for reliable shape-memory actuators?
(3) Are our designed geometries optimal or can we improve them?
(4) Will we manage activation by internal or external, punctual or distributed heating?
(5) Should we resort to activation stimuli different from temperature changes?
(6) Can our system benefit from combining smart materials for multi-stimuli responses?

Devices based on the use of “smart” materials usually include a portion of the “smart” or multi-functional material, as transducer capable of sensing or actuating, within an already designed device or structure, typically using the novel material as substitute for an existing and more conventional sensor or actuator. The use of shape-memory polymers, thanks to their excellent manufacturability and to the possibility of obtaining both simple and complex geometries (as further discussed in Section 2.3), provides additional versatility to the design stage.

Indeed shape-memory polymers can be used, either as a minor active part of a device with a predefined structure, or to develop complete geometrically complex devices. An excellent example is the development of a medical device for treating stroke based entirely on a shape-memory polymer structure with permanent spiral geometry and a temporary linear form, in which the shape-change is activated by light [27]. In other cases, the shape memory polymer is just needed for the active part of the structure, as happens in the multi-pede chair project with a SMP core, surrounded by rubber and supported by aluminum rods [28].

Deciding whether the shape-memory polymer will be used of the whole device structure or just as accessory active element is a relevant issue affecting all subsequent processes. The same decision has to be made when benefiting from 4D printing approaches, as the final device may be made of a passive structure with some active zones, if multi-material printing is employed. The effects of the passive structures as compliant elements, against which the active shape-memory element must perform its actuation, can be modeled with the support of finite-element models if the thermo-electro-mechanical performance (or the response to other stimuli) of the shape-memory polymer is well know after methodic characterizations. In other more complex systems, combinations with other families of “intelligent” materials or transducers may allow designers to reach systems with both sensing and actuation capabilities or even self-sensing actuators, which should be considered from the conceptual design stage, as these functionalities are usually basic product specifications [23].

**Figure 2:**
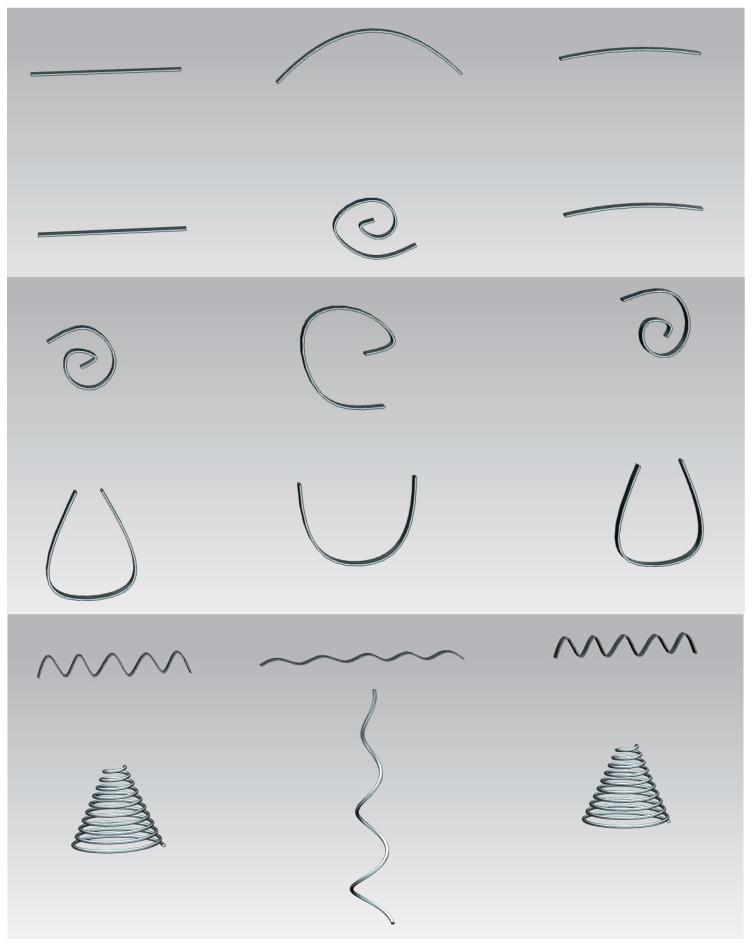
Summary of typical conceptual geometries for reliable actuators based on shape-memory polymers. The left column includes the original geometries, the central column includes the temporary shapes after training and the right column shows the finally recovered shapes.

As support for designers, Figure 2 includes a summary of typical geometries for reliable actuators based on shape-memory polymers. Simple geometries include bending cantilevers, axial rods, linear springs or torsion springs. More complex ones include screw-like actuators, multi-fingered claws, foldable structures and passive structures with expanding or compressing SMP elements, among other possibilities, and can be found in forthcoming sections and in the references.

**Figure 3:**
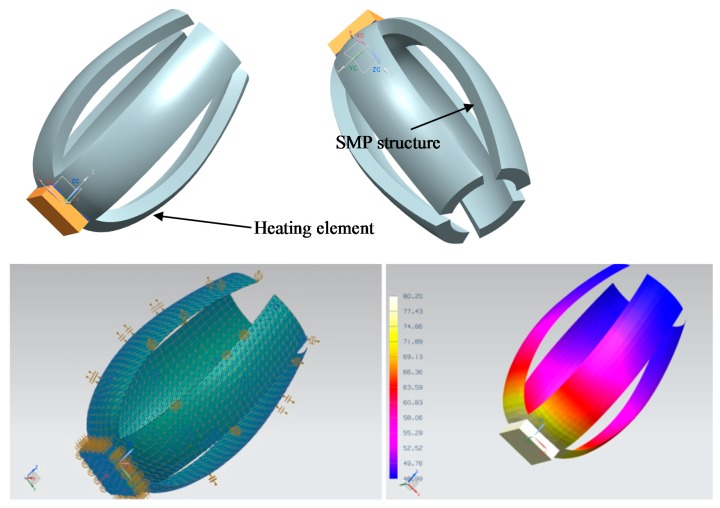
Example of a heating system for SMP activation designed using FEM (finite-element modeling) simulations.

Alternatives with the whole structure based on shape-memory polymers and examples using the shape-memory polymer just as a supporting active zone are possible and complementary approaches, as the example provided in Figure 3 helps to detail. As Figure 2 schematically shows, the shape recovery is not always perfect, especially if the device recovers against external forces. The use of behavior models implemented with the input of exhaustive characterization [29,30] and the support of computer-aided design and engineering resources [31] are good-practices to evaluate the eventual changes to the original shape, as well as the extra deformations required during the training process, so that the recovery leads to the desired final shape. Topological optimization resources are also useful for enhancing the mechanical performance and overall endurance/weight ratio.

Another special design issue, linked to the development of intelligent devices based on the use of shape-memory polymers as actuators, is the need of a heating system for training the shape-memory effect and for activating the shape recovery, in the case of the more common thermo-electro-active SMPs. There is a widespread use of “punctual” distributed heating resistances, working via Joule effect heating of small resistors connected in series, as activation method. In spite of its simplicity and effectiveness, Joule effect heating using resistors involves several issues needing attention: Final device size is normally increased due to the additional space required for the resistances. Furthermore, the use of resistances limits materials’ strength and the obtained devices are normally weaker. Finally, the activation process through heating resistances is not homogeneous, thus leading to important temperature differences among the polymeric structure and to undesirable thermal gradients and related stresses, also limiting the application fields of shape-memory polymers. Alternative actuation mechanisms [12,13,14,15,16,17,18,19,27] can also be modeled resorting to multi-physical/chemical finite element modeling.

In fact, interesting alternatives towards more homogeneous heating are providing solutions to such issues. It is worth mentioning the use of nickel nanoparticles, carbon black, carbon nanotubes, among other possibilities, embedded inside the SMP, in order to obtain electroactive shape-memory polymers, whose heat-based activation process is faster, more controllable and more efficient, as a result of the homogeneous distribution of the heating particles. Additional information on electroactive shape-memory polymers can be found in previous research on the use of carbon nanoparticles [32,33] and on the use of nickel nanoparticles [34,35] (for a review on the topic, see [36]). In more recent studies, the use of nanopapers with embedded nanotubes has been also described [37,38]. Nanopapers may act as coatings for promoting the conductivity of shape-memory polymers and enabling their activation by heat transfer from the nanopaper to the shape-memory polymers, what opens new activation possibilities, highly linked to other alternatives based on the use of electro-textiles [39].

It is important to remark that, in previous devices based on shape-memory polymers, typically activated using heating resistances (as well as in recent studies from our group using Peltier heater-coolers [40]) temperature differences around 30 °C to 70 °C can usually be found within the core of the polymer, while these novel electroactive shape-memory polymers provide temperature differences normally lower than 30 °C among the whole structure. Another interesting possibility is linked to the incorporation of nanoparticles into shape-memory polymeric devices or structures for promoting induction heating, thus enabling remote activation of the shape-memory effect for wireless devices and implants [27,41]. However, the impact of nanoparticle inclusion on the mechanical properties is also relevant and should be addressed, as well as the influence of processing on final device cost. We should not forget that the use of nanocomposites requires systematic synthesizing and processing methods, as well as special security issues linked to working with nanoparticles.

In any case, advanced multi-physics simulation resources, including the FEM (finite-element modeling) thermo-mechanical simulation modules available in different state-of-the-art computer-aided design and engineering programs, prove to be very useful for an adequate design of the activation system of SMP-based devices. Figure 3 includes an example of a heating system for SMP activation designed using thermal FEM simulations. Prototype testing with the help of infrared thermography constitutes an excellent validation procedure for adjusting the simulations for further design activities, as further discussed in Section 2.4.

### 2.3. Challenges and Solutions Linked to the Manufacturing Stage

The manufacturing stage of actuators based on shape-memory polymers also includes particular considerations, which may help to reach more adequate devices. Different discussion questions for a more direct manufacturing stage include:

(1) Are the geometries of the device and actuator simple enough for mass production?
(2) Should we resort to additive manufacturing, both for prototyping and production stages?
(3) How can we integrate the different subsystems of our “smart” actuator?
(4) Can we promote special properties for a more adequate interaction with the environment?

If the actuator geometry is simple enough, we can employ conventional production technologies, such as casting and injection molding, for obtaining the desired shape-memory polymer devices. Open and closed molds can be used, depending on the geometry, material properties and processing issues. If the geometry is more complex, with inner details, porous features or lattice structures, additive manufacturing technologies, many of which work with photo-polymers having shape-memory properties, are the best option [6]. These technologies, including digital light processing and laser stereolithography, were initially aimed at rapid prototyping applications but are increasingly being employed for mass production of final applications, especially if the desired geometries cannot be obtained with traditional processes [10].

Machining is normally not an available choice, as the heat generated during the machining operations affects the properties of polymeric materials. In some cases, rapid tooling processes are used, for producing soft molds after rapid prototyped parts, in which shape-memory polymers with improved performance can be casted [20]. In the case of biomedical applications, bio-photo-polymers can be also manufactured by means of additive processes for obtaining complex actuator geometries [17], but their shape-memory properties still need to be assessed for the promotion of applications.

**Figure 4:**
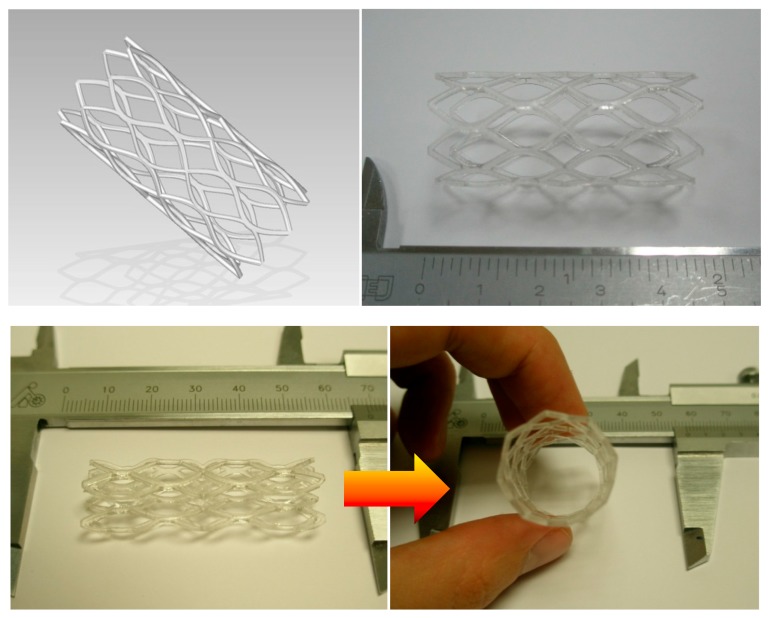
Computer-aided design of the complex geometry of a SMP-based stent, rapid prototype, compressed temporary shape and shape recovery by heating. Adapted from author’s Thesis [10].

By means of example, Figure 4 shows the computer-aided design of the complex geometry of a SMP-based stent, the rapid prototype obtained in epoxy resin by laser stereolithography, the compressed temporary shape after shape-memory training and the shape recovery after heating and consequent activation of the shape-memory effect.

Although epoxy based systems, due to material toxicity, are not adequate for in vitro or in vivo trials aimed at studying the interaction of the devices with living tissues, the use of additively manufactured devices using bio-photo-polymers and bio-photo-elastomers is always a possibility, as already mentioned. Another possibility for the case of preliminary in vitro trials is the use of special post-processes, such as physical or chemical vapor deposition processes, for applying thin coatings upon the surfaces of the actuator. For instance, the use of diamond-like carbon coatings upon epoxy prototypes has proven to be an excellent solution for solving the toxicity issues of epoxy resin for the development of cell culture devices [42].

Some recent studies have also resorted to exhaustive characterization techniques, such as dynamic mechanical analyses, for obtaining full knowledge about the properties of the parts and devices being manufactured by additive technologies using shape-memory polymers. In such studies, photo-polymerizable epoxy resins were also used and the methodic characterization enabled researchers to appreciate the influence of factors including: part geometries, machine precision, processing and post-curing conditions or the use of additives and reinforcements for ad hoc tuning of properties to the final application [43,44,45,46]. The impact of additional factors on the properties of SMPs, especially as regards activation temperature, should also be taken into account and studied. For instance, humidity and physical ageing have proven influential in the appearance of progressive modifications of *T*_act_, which can dramatically affect their performance [9,12,13,14,15]. We would also like to highlight the relevance of carefully analyzing the properties and biological effects of innovative photosensitive materials for stereolithography, usually referred to as “medical resins”, especially if final applications aimed at interacting with the human body, as in the case of active implantable medical devices or special surgical tools, are sought [47,48,49]. All these aspects constitute interesting and innovative research trends for improving the performance and impact of SMP-based actuators.

### 2.4. Challenges and Solutions Linked to the Validation and Operational Stages

The validation and operation stages of actuators based on shape-memory polymers also comprise particular considerations, which may help to reach more adequate responses. Different discussion questions for a better performance include:

(1) Is the conceived shape-memory training process satisfactory or can we improve it?
(2) How can we obtain relevant information during the validation of the shape-memory effect?
(3) Can we improve the long-term performance of our device?
(4) Can we promote security during operation and manage unexpected risks?

Typical procedures for training the shape-memory of a polymeric device, involving the change from the original or “permanent” shape to the temporary geometry, include heating processes using ovens, hot-air guns and immersion in hot water. When the polymer goes beyond its activation temperature, the geometrical changes are imposed and sudden cooling leads to the temporary geometry. In some cases, the use of the already designed heating system with the help of control electronics can be used for a more controlled training process. Such control relies on an adequate evaluation of the temperature field within the “smart” actuator, which can be performed with the help of infrared thermography to promote security and to avoid unexpected temperature increases, which may damage the material or change its special properties.

Similar processes can be used for testing the shape-memory effect and the recovery process towards the initial or “permanent” shape. The use of infrared thermography, apart from providing interesting information regarding the actuator’s performance, also promotes security during operation and limits the possibility of accidents, helping also to detect and manage unexpected risks. The use of these tools is easier than monitoring the whole device with thermocouples and temperature sensors, which may affect actuator’s performance. Finally, it is necessary to point out that infrared thermography, being a non-destructive test, helps to promote a sustainable and cost-efficient development process.

**Figure 5:**
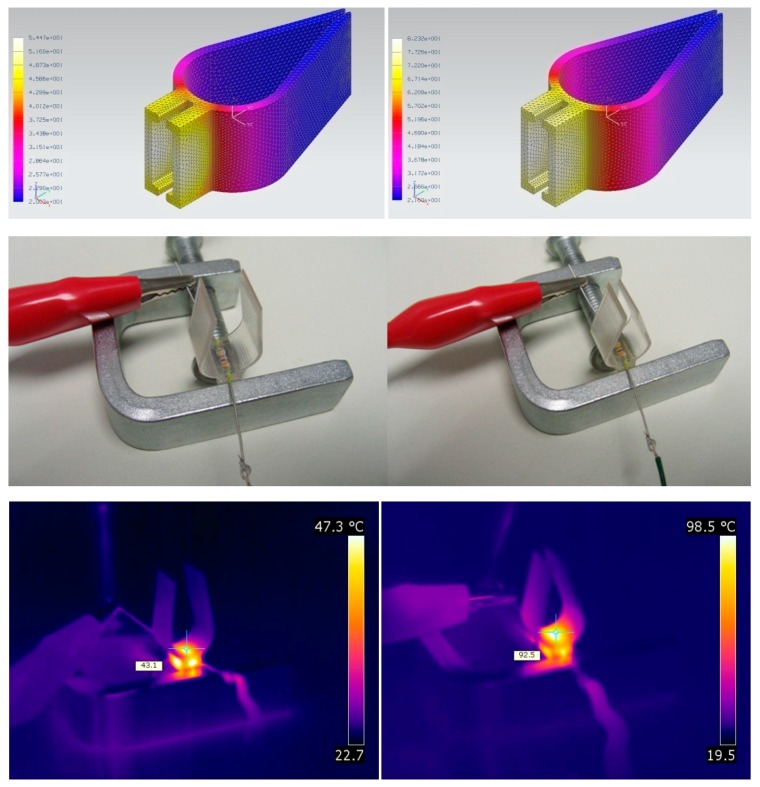
Validation of the activation system of a SMP surgical pincer: Design carried out with the help of thermal finite-element simulations and validation supported by IR-thermography for comparative purposes. See [10,21] for additional details regarding the use of FEM modeling and IR-thermography in the development of smart actuators based on shape-memory polymers.

For example, Figure 5 shows the validation of the activation system of a SMP surgical pincer, including images of the design carried out with the help of thermal FEM simulations and of the validation step supported by infrared thermography for comparative purposes. In such development, infrared thermography is also employed to validate the thermal simulations (in a very direct and easy to understand manner), which can then be used for further developments and for final design optimization tasks, with the additional confidence provided by having a quantitative evaluation of simulated functionalities.

In many cases, the geometrical changes of a shape-memory polymer based “smart” actuator are quite simple, as schematically shown in some of the examples of Figure 2. Bending, stretching or twisting a shape-memory stripe can be usually performed with very simple equipment or mechanical testing units. The use of thermally controlled mechanical testing machines, not just for characterizing the shape-memory polymer, but also for obtaining the temporary shape, has been previously highlighted [8]. 

**Figure 6:**
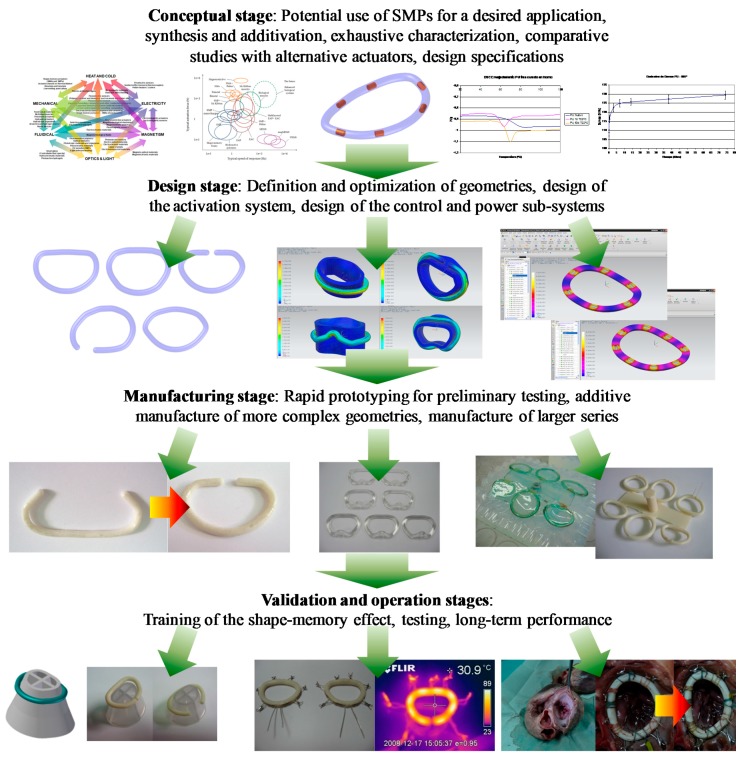
Summary of the complete development process, supported by computer-aided engineering and additive manufacturing approaches, for a “smart” annuloplasty ring based on shape-memory polymers oriented to the potential treatment of mitral valve insufficiency. Adapted from author’s Ph.D. Thesis [10].

However, in some cases, special resources and tools are required for changing from the original to the temporary shape. The use of ad hoc designed geometries for hot-pressing the shape-memory actuator may be a good option for training the shape-memory effect. Such geometries can be again rapidly obtained by resorting to rapid prototyping technologies, normally based on additive manufacturing approaches. An example of a rapidly developed shape-training work bench is included in the final validation stage of the development process shown in Figure 6 and further detailed in the following summary Section 3.

Regarding long-term performance, it is important to characterize, and take into account, from the design stage, the impact of physical and chemical ageing processes on the shape-memory properties and activation temperatures of the developed actuators. Aspects such as humidity and temperature during the transport and storage of already trained geometries with shape-memory must be also considered, as they could even promote the activation of the shape-memory effect before the desired operation stage. A relevant feature of some SMPs, studied and developed recently for improving their long-term performance, is their potential self-healing ability, which tries to mimic the interesting performance of biological materials. Foundational studies have pursued this by rational molecular design of the polymeric chains, networks and bonds [50,51].

Towards the industrial success of these smart actuators, which normally work as subsystems within more complex devices, it is important to also take into account the assembly and disassembly operations (i.e., for enabling maintenance activities or for achieving innovative performances) and their impact on the designed geometries, which should be oriented, even from the conceptual design stage, to such assembly and disassembly processes. Key aspects linked to these operational topics have been lately analyzed in a systematic way [52].

In terms of environmental issues affecting the success of SMPs in the operational stage, it is necessary to pay attention to the biological performance of shape-memory polymers used for medical devices and applications. This should be considered from the conceptual and design stages, although possibly some specific aspects of these interactions between materials and tissues can only be found along the validation and operation stages, during in vitro or in vivo animal trials aimed at verifying devices’ compliance with the regulatory directives and standards. Much has been achieved in recent years in terms of improved chemistries for the enhanced biological response of SMPs and for their being able to perform shape changes at body temperatures. The more interesting shape-shifting effects, working mechanisms and materials for biomedical uses (which have been recently reviewed [53]) provide researchers in the field with several possibilities, as well as with innovative research directions.

Section 3 summarizes all the aforementioned issues and proposed good-practices, in the form of check-list for developers of “smart” actuators based on shape-memory polymers.

## 3. Summary and Discussion of Strategies for the Straightforward Development Methodology

This section includes a summary of the aforementioned design challenges, typical good-practices and potential solutions for the systematic development of reliable, effective and efficient “smart” actuators based on shape-memory polymers. Figure 6 includes a summary of the proposed complete development process, supported by computer-aided design and engineering resources and by additive manufacturing technologies for a more systematic and direct path to the operation stage. The selected case study is linked to the complete development process of a “smart” annuloplasty ring, based on shape-memory polymers and oriented to the potential treatment of mitral valve insufficiency, from the conceptual design stage to the in vitro testing phase. Such a device is aimed at implantation according to the expanded (and insufficient) valve geometry and at a subsequent step-by-step and progressive actuation, leading to a compressed geometry for the promotion of adequate valve closure, after the patient recovers from the initial surgical procedure.

In addition, Table 1 and Table 2 include main challenges and potential solutions in the conceptual, design, manufacturing and operation stages typical of the development process of “smart” actuators and systems based on shape-memory polymers. Relevant references, with additional cases of success, are included in the right columns for further details regarding some special solutions, which hopefully prove useful for researchers, developers and innovators working in the field of smart materials, structures and devices. These tables may provide designers with a rapid process to detect important design issues, when developing SMP-based smart engineering systems, and a very direct reference to interesting examples of good practices, developed by relevant researchers in the field of smart materials, which may support designers in their approach to using SMPs as transducers for intelligent devices. After presenting the tables, a final discussion regarding the potentials of SMPs, as materials for the development of “smart” actuators, and analyzing their benefits and drawbacks as members of the large family of multi-functional materials is included.

As summarizing reflection, it is important to highlight that shape-memory polymers, as family of multi-functional materials, are quite novel and all aspects of the life-cycle of innovative products based on shape-memory polymers have very importantly evolved in the last two decades. The synthesis strategies and processing techniques, the characterization and testing facilities, the design and modeling resources and the prototyping and manufacturing tools, available for the creation of new concepts and actuators based on SMPs, are continuously evolving. In addition, highly innovative and extremely powerful features, which promote the versatility of these smart polymers, have been developed recently, including their multi-step actuation possibilities and their self-healing performances. All these advances and innovations provide designers with an enlarged freedom of creation but, at the same time, increase the need for well-trained professionals and add uncertainties to the life-cycle of products based on advanced materials still under continued development and improvement. 

Furthermore, the already large family of multi-functional materials continuously works and SMPs compete with shape-memory alloys, with electro-active polymers and with biofabricated active materials, to cite some examples. Understanding the limits of SMPs and their potential synergies with other “smart” materials for the development of more effective and efficient intelligent engineering systems is thus a requisite for successful designers (and designs). Consequently, counting with a methodic summary of challenges and good practices, as the one presented here, which can be continuously updated and may lead to the integration of design strategies or to a sort of systematic development methodology, is relevant to help novel designers in their approach to the marvels of shape-memory polymers.

## 4. Conclusions

A straightforward development of “smart” actuators and “intelligent” devices, based on the use of shape-memory polymers, requires additional emphasis on the application of systematic development methodologies. The present study has presented a summary of challenges and good practices, leading to a straightforward methodology or integration of strategies, for the development of “smart” actuators based on shape-memory polymers. A “Conceive–Design–Implement–Operate” (CDIO) approach has been proposed and main challenges, potential solutions and current trends of research, linked to the different stages, have been discussed. Based on the experience acquired along the development of different (bio-)devices, taking advantage of the remarkable properties of shape-memory polymers, more than thirty challenges or relevant design issues, linked to the conception, design, implementation and operation stages have been summarized, together with related good-practices and proposals for efficient solutions. Some relevant questions still needing an answer have been also analyzed and discussed, while presenting also some directions for future research following the steps of groundbreaking researchers and their reviewed strategies.

## Figures and Tables

**Table 1:** Main challenges and solutions in the conceptual and design stages for the development of “smart” systems based on shape-memory polymers.

Main Stages	Sub-Stages	Common Challenges	Typical Good-Practices and Potential Solutions	Refs.
Conceptual	Actuation principle	Select the most adequate “smart” material for a novel “smart” actuator	Use “Ashby” charts focused on “smart” materials and structures	[23]
Conceptual	Actuation principle	Assess if using a SMP is adequate for the required levels of strain and stress	Use “Ashby” charts focused on SMPs and data from publications	[23]
Conceptual	SMP selection	Obtain all the relevant information for the design stage	Exhaustive characterization, besides existing data sheets	[8,43,44,45,46]
Conceptual	SMP selection	Obtain all the relevant information for the manufacturing stage	Carry out preliminary tests, besides existing data sheets	[8,43,44,45,46]
Conceptual	SMP selection	No existing SMP seems to fulfill the actuation requirements	Change from actuation principle or use another active material, if the mismatch is relevant, or synthesize a new one or use additives for existing ones, if the mismatch seems salvable	[1,2,3,7,22,23]
Conceptual	Specifications	Provide all the relevant requirements and expected properties, regarding the SMPs actuators, for the designers	Proceed as with conventional off-the-shelf engineering actuators but taking account of long-term performance, chemical stability and toxicity, and other issues affecting polymeric materials	[8,43,44,45,46]
Design	Define geometries	Define overall geometries for a need	Use computer-aided design even in a personalized way	[20,21]
Design	Optimization	Adapt geometries to requirements	Use computer-aided engineering (mechanical simulations)	[20,21]
Design	Optimization	Define the active area of the device	Use computer-aided engineering (mechanical simulations) and SMP behavior models	[20,21]
Design	Activation system	Design the activation system	Use computer-aided engineering (mainly thermal, electro-magnetic or fluidic simulations)	[20,21]
Design	Activation system	Reduce the size of the activation system (eliminating heating resistors or shifting to other triggering stimuli)	Use distributed heating wires, (nano-)particles, SMP composites, laser-assisted or even water-assisted or chemical-based activation	[27,32,33,34,35,36,37]
Design	Activation system	Promote uniform temperature (suppressing punctual heaters)	Use distributed heating wires, (nano-)particles or SMP composites	[32,33,34,35,36,37]
Design	Activation system	Activate in a distant wire-less way	Use induction heating	[41]
Design	Activation system	Promote multi-stepped actuation	Use triple shape-memory materials, Peltier activation, 4D printing or combine with other “smart” materials	[16,17,18,19,24,25,26]
Design	Control and power	Design the control and power supply sub-systems adequately	Evaluate the thermal loads with the help of FEM resources	[20,21]
Design	Control and power	Promote space savings by eliminating embedded power supply sub-systems (i.e., eliminate batteries and wires)	Supply power in a remote way using inductive approaches or near field communication	[41]

**Table 2:** Main challenges and solutions in the manufacturing and validation stages for the development of “smart” systems based on shape-memory polymers.

Main Stages	Sub-Stages	Common Challenges	Typical Good-Practices and Potential Solutions	Refs.
Manufacture	Prototyping	Obtain rapid prototypes for testing	Use additive manufacturing	[10,20]
Manufacture	Prototyping	Achieve complex geometries	Use additive manufacturing	[10,20]
Manufacture	Prototyping	Prototyping of simple geometries	Use rapid molds made of spare components and simple geometries	[10,20]
Manufacture	Larger series	Obtain prototype series for testing	Additive manufacturing or rapid-form copying in PDMS molds	[10,20]
Manufacture	Larger series	Achieve complex geometries	Industrial additive manufacturing	[10,20]
Manufacture	Larger series	Large series of simple geometries	If possible extrusion processes or injection molding	[10]
Manufacture	Larger series	Minimize costs while improving performance	If possible extrusion or injection molding, use SMP composites	[10]
Manufacture	Post-processing	Integration with surrounding sub-systems or with existing devices	Consider connections from the design stage and use off-the-shelf connecting components	[10]
Manufacture	Post-processing	Promote special surface phenomena (biocompatibility, tribological properties, long-term endurance)	Use low-temp. chemical/physical vapor deposition processes for incorporating special features without affecting the SMP	[38,39,42]
Validation and operation	Training of shape-memory effect	Obtain the desired temporary shape if complex changes are involved	Use rapid prototyped work-benches and support geometries	[10,20]
Validation and operation	Training of shape-memory effect	Promote a homogeneous temperature during training for enhanced effects	Control the process with IR thermography and sensors and compare with thermal simulations for improved design strategies	[21]
Validation and operation	Fulfillment of specifications	Validate if the developed stresses during actuation and the deformations obtained are according to specs	Control the process with sensors and stress/strain-measuring cameras and compare with mechanical simulations	[21]
Validation and operation	Fulfillment of specifications	Validate if the temperatures during actuation are according to specs	Control the process with IR thermography and sensors and compare with thermal simulations	[21]
Validation and operation	Long-term performance	Prevent changes to the SMP activation temperature	Take into account the effects of chemical and physical ageing and the impact of environment from the design stage	[9,12,13]
Validation and operation	Long-term performance	Consider impacts of time and environment on the SMP performance	Take into account the effects of chemical and physical ageing and the impact of environment from the design stage	[9,12,13]
Validation and operation	Assembly and disassembly (i.e., for maintenance)	Integration with surrounding sub-systems or with existing devices	Follow design oriented to assembly and disassembly principles and the right geometries and components	[52]
Validation and operation	Reparation of damaged features	Minimize maintenance tasks and promote long-term performance by taking benefit from shape changes	Take advantage of new development linked to self-healing SMPs	[50,51]
Validation and operation	Security issues	Prevent excessive temperature values during the activation	Control the values using self-sensing approaches or using paramagnetic-diamagnetic transitions of nanoparticles	[10,41]
Validation and operation	Security issues	Detect and manage risks during actuator service	Resort to self-sensing or even self-healing design approaches	[10,41]
